# Illuminating Biomimetic Nanochannels: Unveiling Macroscopic Anticounterfeiting and Photoswitchable Ion Conductivity via Polymer Tailoring

**DOI:** 10.1021/acsnano.4c08801

**Published:** 2024-09-20

**Authors:** Yi-Fan Chen, Vaishali Pruthi, Lin-Ruei Lee, Yu-Chun Liu, Ming-Hsuan Chang, Patrick Théato, Jiun-Tai Chen

**Affiliations:** † Department of Applied Chemistry, National Yang Ming Chiao Tung University, 300093 Hsinchu, Taiwan; ‡ Institute for Chemical Technology and Polymer Chemistry (ITCP), Karlsruhe Institute of Technology (KIT), Kaiserstraße 12, D-76131 Karlsruhe, Germany; § Soft Matter Synthesis Laboratory Institute for Biological Interfaces III, Karlsruhe Institute of Technology (KIT), Hermann-von-Helmholtz-Platz 1, D-76344 Eggenstein-Leopoldshafen, Germany; ∥ Center for Emergent Functional Matter Science, National Yang Ming Chiao Tung University, 300093 Hsinchu, Taiwan

**Keywords:** anodic aluminum oxide, anticounterfeiting, biological nanochannel, ion conductivity, photoswitchable, spiropyran

## Abstract

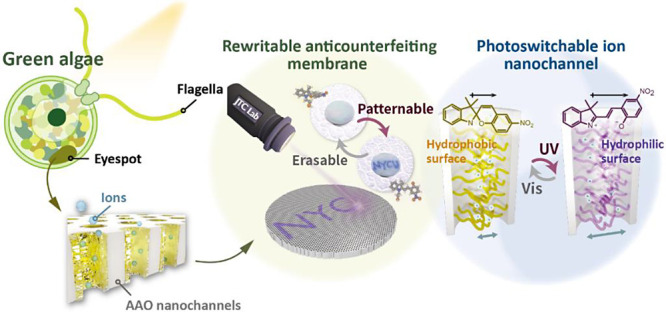

Artificial photomodulated channels represent a significant advancement toward practical photogated systems because of their remote noncontact stimulation. Ion transport behaviors in artificial photomodulated channels, however, still require further investigation, especially in multiple nanochannels that closely resemble biological structures. Herein, we present the design and development of photoswitchable ion nanochannels inspired by natural channelrhodopsins (ChRs), utilizing photoresponsive polymers grafted anodic aluminum oxide (AAO) membranes. Our approach integrates spiropyran (SP) as photoresponsive molecules into nanochannels through surface-initiated atom transfer radical polymerization (SI-ATRP), creating a responsive system that modulates ionic conductivity and hydrophilicity in response to light stimuli. A key design feature is the reversible ring-opening photoisomerization of spiropyran groups under UV irradiation. This transformation, observable at the molecular level and macroscopically, allows the surface inside the nanochannels to switch between hydrophobic and hydrophilic states, thus efficiently modulating ion transport via changing water wetting behaviors. The patternable and erasable polySP-grafted AAO, based on a controllable and reversible photochromic effect, also shows potential applications in anticounterfeiting. This study pioneers achieving macroscopic anticounterfeiting and photoinduced photoswitching through reversible surface chemistry and expands the application of polymer-grafted structures in multiple nanochannels.

## Introduction

Within addressable nanodevices, light-responsive systems distinguish themselves by their capacity to transform optical signals into a range of useful output signals. These signals are essential in applications in diverse fields, including sensors,^1^ photogated delivery,^2^ optical information storage,^3^ and biomedical engineering.^4^ Caused by their high sensitivity, remote controllability, and reversibility, light-responsive materials have been investigated not only on a macroscopic scale but also applied on much smaller scales—such as microelectromechanical systems^5,6^ or even in nanoconfined environments^7−9^—that can hardly be influenced by other stimuli. In such systems, the incident light triggers either photomechanical deformation or polarity inversion, which can furnish confined environments with different physicochemical properties.^10,11^

To construct such photomodulated structures, several materials are applied based on different structural designs. As photoresponsive moieties, azobenzene or spiropyran is often used because of its molecular isomerization upon light irradiation. This photoinduced transformation widely impacts microstructures, i.e., polarity and molecular length, as well as macroscopic characteristics, including hydrophobicity, photochromism, and motions.^12−14^ Aiming to enhance the sensitivity of the photoresponsive moieties, nanomaterials with versatile shapes, such as polymer brushes,^15^ anisotropic nanoparticles,^16^ polymer films,^17,18^ and single conical nanochannels,^19,20^ have also been studied due to their high surface areas. Especially channel structures, which are similar to the respiratory system and membrane transport, create confined environments to simulate both interactions and reactions inside.^21−26^

Taking a look into nature, photomodulated biological channels activated via light irradiation serve specific functions related to the regulation of physiological phenomena or cellular processes.^27,28^ Acquiring insights from nature, several studies have explored the creation of artificial nanochannels controllable by light for diverse applications.^29,30^ For instance, guard cells in stomata allow gas exchange and water vapor loss via reversible movements, which inspired either structural designs or controllable mechanisms in previous research.^31,32^ In terms of the porous light-harvesting systems, metal–organic frameworks (MOFs) anchored with multiple pigments have been reported to understand the charge/energy transfer process.^33,34^ Last but not least, versatile channel-based gating based on the concepts of photoreceptors for phototaxis has been developed to achieve engineered biological valves, which are relatively challenging missions owing to the limited environments for photoinduced conformation transformation.^35,36^

The artificial photomodulated channels represent a significant advancement toward practical photogated systems because of their remote noncontact stimulation. Yet, ion transport behaviors in artificial photomodulated channels still require further investigation, especially in multiple nanochannels that closely resemble biological structures. Based on this, we herein explore the photoinduced ion transport behaviors in multiple nanochannels by grafting photoswitchable spiropyran polymers in nanoporous anodic aluminum oxide (AAO) nanochannels.

To mimic the multiple ion nanochannels in the cell membranes of green algae, we select AAO membranes as substrates because they also serve functions by constructing high surface areas and vertically aligned interfaces. Meanwhile, polymer structures are also considered to imitate biomacromolecules, providing a higher density of photoreceptors than monolayers. As photoresponsive moiety, spiropyran was used in this study as photoreceptor in the ion channels. By modification of polymerizable methacrylate functional groups on spiropyran (SPOH), a spiropyran monomer (SPMA) was prepared. To enhance the reactive areas and suppress the massive aggregation of the spiropyran moieties, we applied surface-initiated atom transfer radical polymerization (SI-ATRP) to tether spiropyran-containing polymers (polySP) on the surfaces of AAO nanochannels.

By light irradiation, the reversible ring-opening isomerization of the spiropyran groups can be induced, resulting in changes in the packing and polarity of the polymer chains. These isomerizations are visible to the naked eye with a color change—from optically transparent to dark purple—which provides an exciting opportunity to track the transformation on a macroscopic scale. Thus, patternable and erasable polySP-grafted AAO membranes based on the controllable and reversible photochromic effect are used to demonstrate their potential application in anticounterfeiting. Taking advantage of the photoactive properties of the nanochannels, we also investigated light-controlled ion transportation in confined environments inspired by the light-gating mechanisms of ChR ion channels. By addition of aqueous electrolytes selected to simulate cellular environments, hydrophilicity and ion conductivity can be reversibly controlled for multiple cycles. This study not only demonstrates photoresponsive polymer-grafted structures in multiple nanochannels but also achieves both macroscopic anticounterfeiting and photoinduced ion photoswitching through reversible surface chemistry, distinguishing itself from various applications based on molecules or single channels.

## Results and Discussion

**Figure 1:**
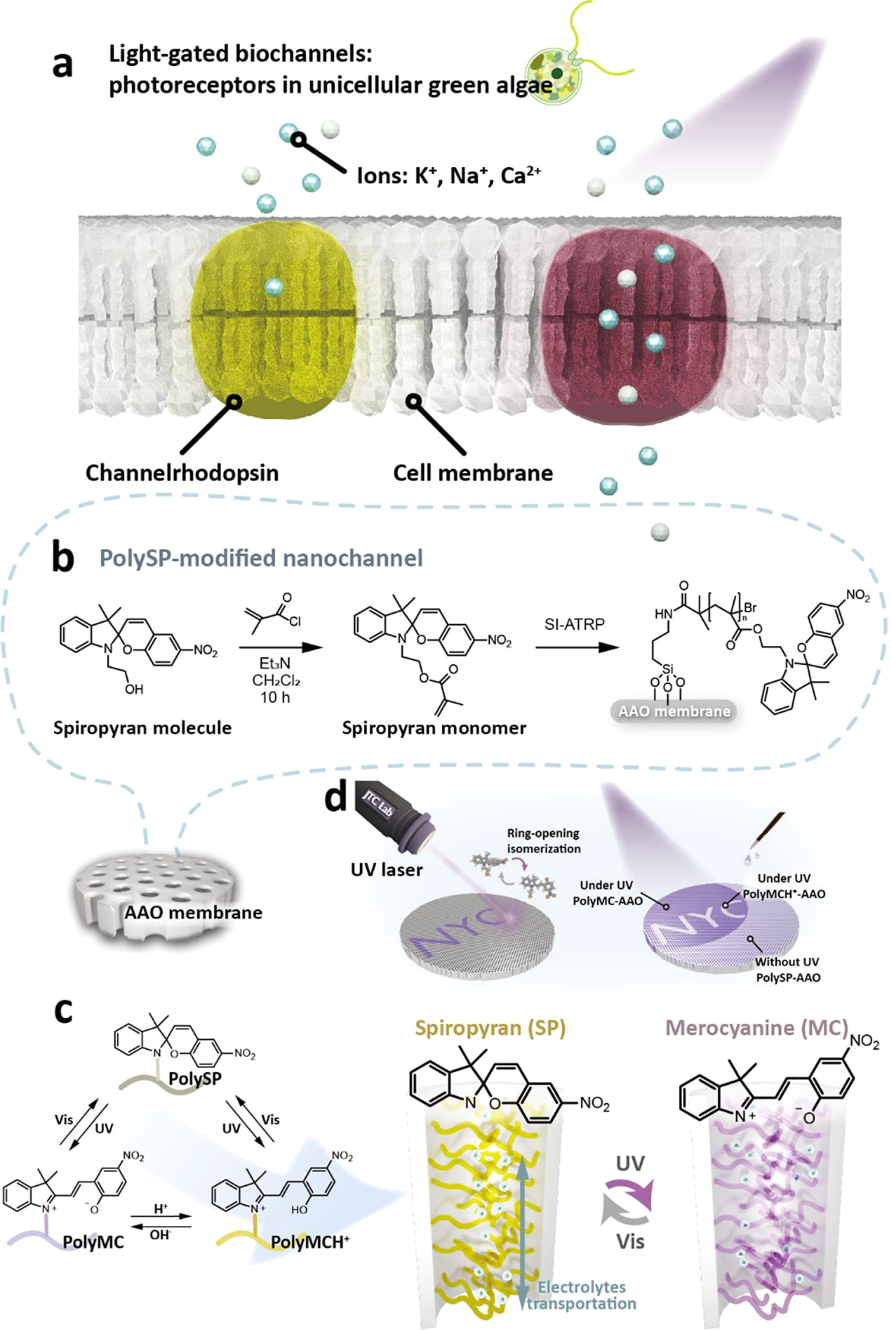
Conceptual design and mechanism illustration of polySP-modified photoswitchable ion nanochannels. (a) Graphical illustrations of biological ChR gates. Opening and closing operations are triggered via irradiation. (b) Schematic illustration of the spiropyran molecule, spiropyran monomer, and polySP-grafted nanochannel. (c) Graphical illustrations of the photoswitchable polySP system, in which the hydrophilicity and on-demand ionic conductivity can be controlled via light irradiation. (d) Graphical illustrations of rewritable and patternable anticounterfeiting polySP-AAO membranes.

As essential producers in aquatic ecosystems, green algae form the foundation of marine food webs and actively participate in carbon sequestration and oxygen production through photosynthesis.^37,38^ To optimize exposures to light for these processes, during which they convert light energy into chemical energy, phototactic responses enable green algae to navigate the environment and maximize access to light. Light-gated ion channels in the cell membranes, known as channelrhodopsins (ChRs), play a crucial role in regulating cellular activity in response to light conditions for optimal photosynthesis and movement,^39^ as displayed in Figure 1a. Upon light irradiation, especially blue or purple light, the ChRs undergo a conformational change to allow the passage of charged ions, which inspired the conceptual design of this work.

The schematic illustration of the photogating molecules and polymers in the ChR-inspired ion nanochannels is displayed in Figure 1b. To mimic the multiple ion nanochannels in the cell membranes of green algae, we select AAO membranes as substrates, which also serve functions by constructing high surface areas and vertically aligned interfaces. Meanwhile, polymer structures are often considered to imitate biomacromolecules, which can provide a higher density of photoreceptors than monolayers. Spiropyran is used as the photoresponsive moiety in this study as a photoreceptor in the channels. Various methods, including thermal annealing,^40^ solution wetting,^41^ solvent vapor annealing,^42^ and direct polymer grafting,^43,44^ have been considered to introduce responsive polymers into AAO nanochannels. Prior research indicates that the spiropyran molecules tend to aggregate with their isomers, leading to the photofatigue effect.^45^ Consequently, we designed polymer grafting as a preventive measure against this occurrence. Spiropyran monomers (SPMA) were fabricated by modifying spiropyran molecules (SPOH) with polymerizable methacrylate functional groups. To enhance the reactive areas and suppress the massive aggregation of the spiropyran moieties, we applied surface-initiated atom transfer radical polymerization (SI-ATRP) to tether the spiropyran-containing polymers on the surfaces of the AAO nanochannels.

Upon UV irradiation, the reversible ring-opening isomerization in different pH values of the spiropyran groups was induced, as presented in Figure 1c. As reported by previous studies,^46,47^ UV light absorption allowed the spiropyran molecules to transform to the open-ring merocyanine-isomer via heterolytic C–O bond cleavage with a dipole moment (μ) change from ∼4.3 to ∼17.7 D.^48,49^ This difference of dipole moment also altered the hydrophobic nature of spiropyran to the hydrophilic surface of merocyanine. In addition, these isomerizations were visible to the naked eye—from optically transparent to dark purple—which provided an exciting opportunity to track the transformation on a macroscopic scale.^50^ The visible color change in selective areas could be demonstrated using short-visible light laser and acid modifications, showing the potential applications in information encryption and anticounterfeiting, as displayed in Figure 1d.

After applying the SI-ATRP technique on the AAO membranes, we then demonstrated light-controlled ion transportation in confined environments, which was inspired by the light-gating mechanism of ChR ion channels. In our system, an aqueous electrolyte (potassium chloride, KCl) was selected to simulate the cellular environments. While exposed to UV light, the polySP-grafted AAO membrane absorbed the light, causing cleavage of the heterolytic C–O bonds in spiropyran polymers and resulting in the formation of zwitterionic merocyanine structures. The hydrophilic surfaces of these highly polar merocyanine structures promote the creation of a stable hydration layer around the ions, thereby enhancing ion transportation across the nanochannels. Upon cessation of UV light, the merocyanine structures gradually reverted to spiropyran through ring-closing isomerization, creating a water-exclusion environment that hinders ion mobility. These light-induced reversible conductance ion nanochannels have several benefits, such as facile fabrication, prompt reaction time, well-regulated reproducibility, and an unobvious photofatigue effect, which can be applied to sensors, photogate delivery, and optical information storage.

**Figure 2:**
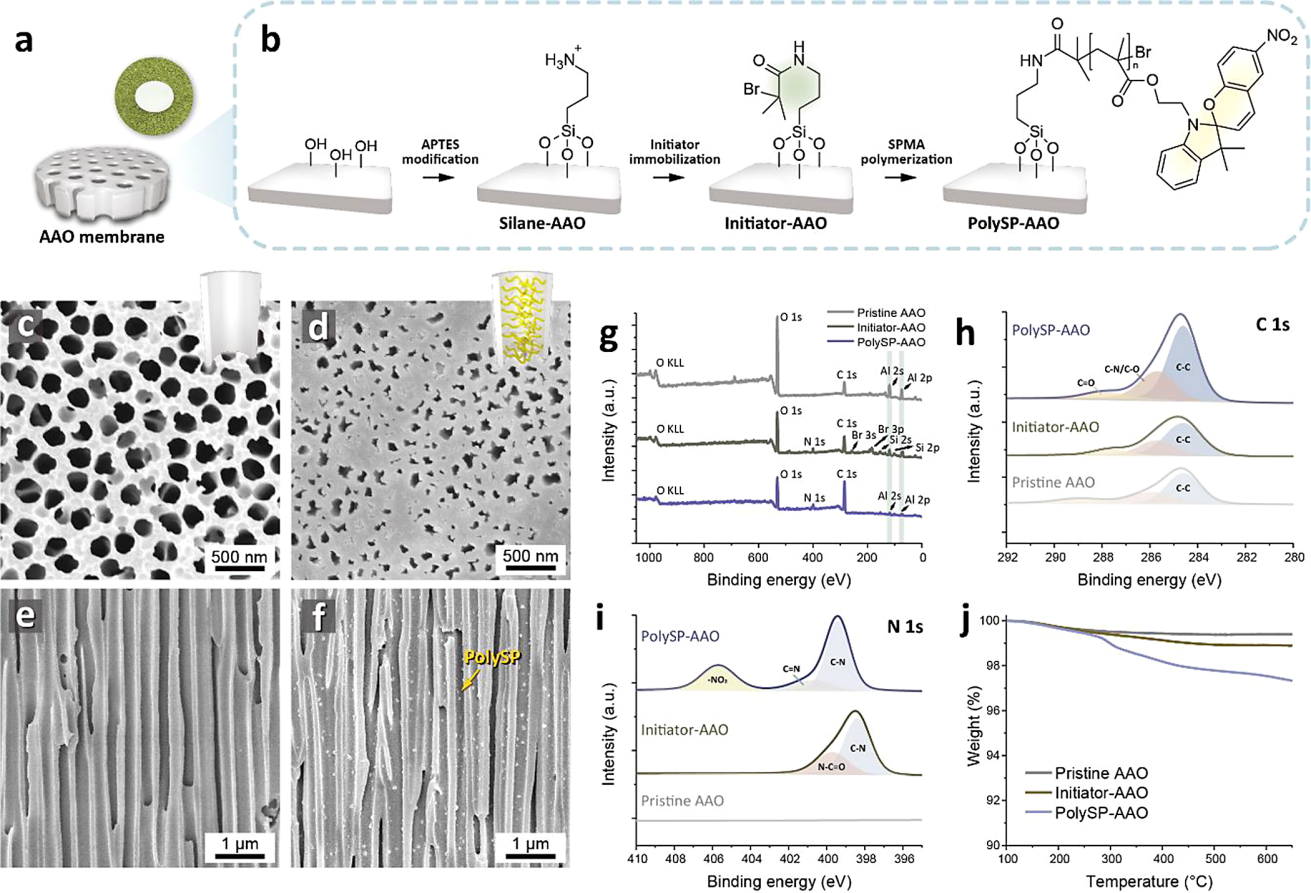
Characterizations of the polySP-modified photoswitchable ion nanochannels. (a) Photograph and graphical illustration of AAO nanochannels. (b) Schematic description of the modification procedures. (c,e) SEM images of the pristine AAO membranes: (c) top-view and (e) side-view. (d,f) SEM images of the polySP-modified AAO membranes: (d) top-view and (f) side-view. (g-i) XPS spectra: (g) survey, (h) C 1s scans, and (i) N 1s scans of the pristine AAO, initiator-immobilized AAO, and polySP-grafted AAO. (j) TGA curves of pristine AAO, initiator-immobilized AAO, and polySP-grafted AAO.

Figure 2a shows a photograph and graphical illustration of the AAO nanochannels, the main reactor, and the container in the following reaction. The polySP-modified photoswitchable ion nanochannels were fabricated by the surface-initiated atom transfer radical polymerization (SI-ATRP) reaction inside the nanopores of the AAO membrane, as displayed in Figure 2b. To stabilize the surface initiators (2-bromo-2-methylpropionyl bromide, C_4_H_6_Br_2_O), oxysilanes containing amino groups were first introduced to the hydrophilic surfaces of the hydroxy-rich AAO nanochannels. Then, the embellishment of the surface initiators was achieved by alkylation of alkyl halides, followed by application of the SI-ATRP reaction to tether spiropyran-containing polymers (polySP) on the AAO nanochannels.

The different nanopore morphologies and porosities were observed, as shown in Figure 2c–f. Figure 2c,d displays the top-view SEM images of the pristine AAO and polySP-grafted AAO nanochannels, respectively. The initial average pore size and porosity of the pristine AAO nanopore were ∼230 nm and ∼40%, respectively. After the polySP modification process, most of the AAO surface was covered with spiropyran polymer. However, the nanopores still existed, with the pore diameter decreasing to approximately 130 nm and the porosity changing to about 16%. Furthermore, the cross-sectional SEM images of the pristine AAO and polySP-grafted AAO nanochannels, as displayed in Figure 2e,f, indicated that the dispersed spiropyran polymer clusters were randomly grown on the side walls of the AAO nanochannels. As a result, the average pore sizes in the nanochannels exhibited a smaller pore size difference from ∼237 to ∼186 nm after the polymer grafting process. Figure S6 presents side-view SEM images at lower magnifications, indicating that the clusters are located throughout the entire nanochannels. EDS mapping and line scan were used to characterize the elements of the interfacial region in polySP-AAO, as presented in Figures S7 and S8. The detailed atomic ratios are also shown in Table S1.

X-ray photoelectron spectroscopy (XPS) was carried out to further verify the grafting of the spiropyran polymers, as shown in Figure 2g–i. On the whole, the peaks at 74.6, 118.1, and 531.7 eV were attributed to Al 2p, Al 2s, and O 1s of the AAO membranes (Figure 2g), respectively. Compared with the pristine AAO, the initiator-AAO showed peaks at 256.5 and 183.5 eV, which were assigned to Br 3s and Br 3p peaks, respectively. The intensities of C 1s peaks increased in both initiator-AAO and polySP-AAO, indicating the successful grafting of the initiators and polymers. Figure 2h shows that the C = O peaks shift from 288.9 to 287.9 eV in pristine AAO and polySP-AAO, which is caused by the addition of valence electrons and increased electron density, suggesting the formation of the polymer brushes. In the N 1s scan, as shown in Figure 2i, polySP-AAO exhibited the peaks at 405.7 and 400.7 eV, which can also confirm the appearances of the nitro group and pyrroline in the spiropyran polymers.

The thermogravimetric analysis (TGA) curves in Figure 2j revealed the decomposition processes of the pristine AAO, initiator-AAO, and polySP-AAO. For the pristine AAO, almost no weight loss was observed at the ramping temperature range of 100–650 °C. As for initiator-AAO, the primary degradation process occurred at ∼200–500 °C, depicting that ∼1% of initiators over the whole AAO membrane were grafted. For polySP-AAO, however, a two-stage degradation process occurred at 300–500 and 500–650 °C, revealing that the grafted spiropyran polymers inside polySP-AAO made up to ∼2.5% compared with the pristine AAO. The differential scanning calorimetry (DSC) measurements of polySP-AAO, however, were difficult because of the thermal contact issue and sensitivity of the instrument.

**Figure 3:**
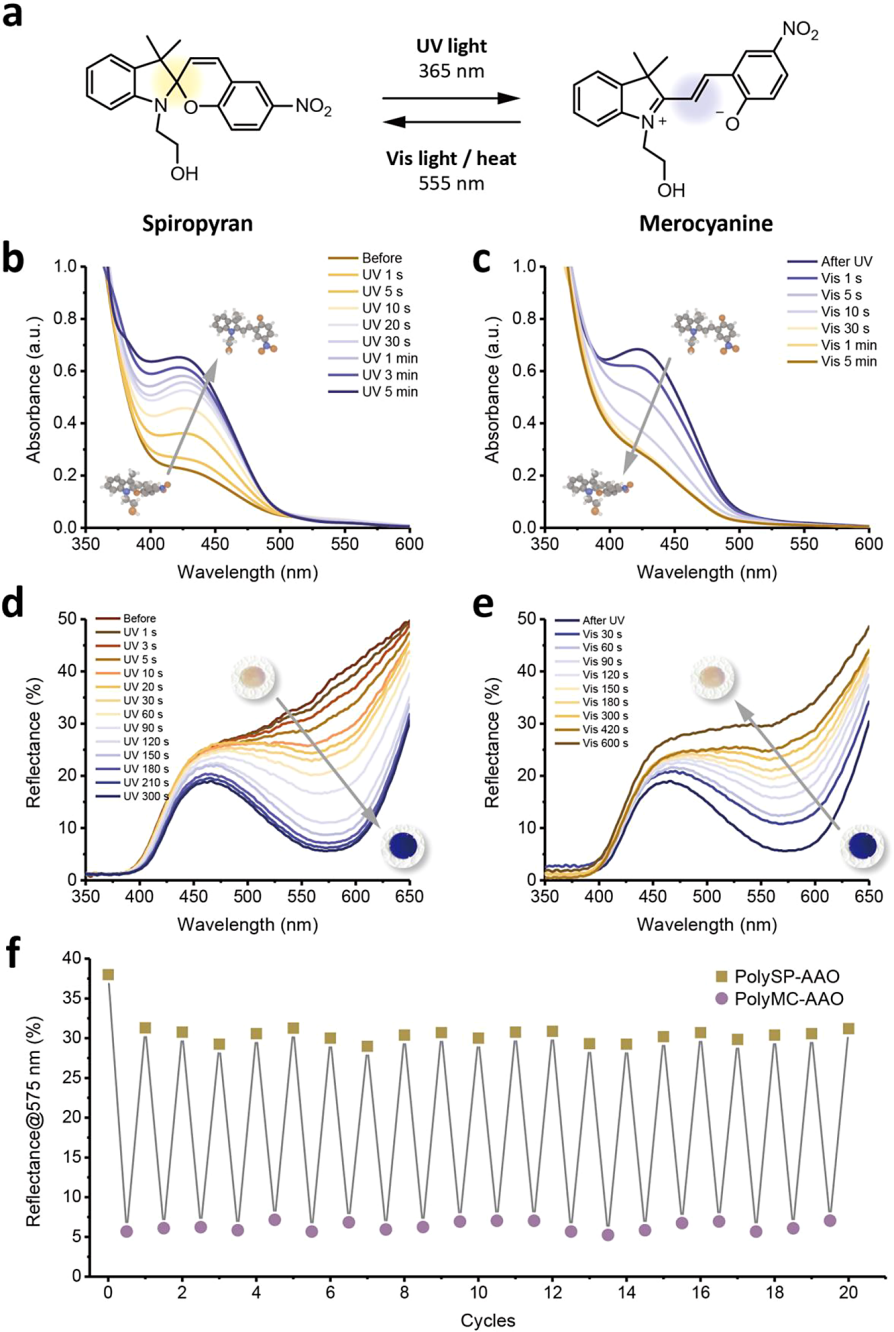
Photoswitching of the ring-opening reaction of the spiropyran molecules and polymers. (a) Reversible ring-opening reaction mechanism of the spiropyran derivative (SPOH). (b,c) UV–vis absorption spectra of the SPOH solutions (b) during UV and (c) visible light irradiations for different times. (d,e) Reflectance spectra and photographs of polySP-modified nanochannels (d) during UV irradiation and (e) visible light irradiations for different times. (f) Multiple ring-opening isomerization of the polySP-modified nanochannels as monitored by recording 20 cycles.

According to the previous studies,^51,52^ the photochemical properties of isomerization of spiropyran molecules upon UV irradiation have been reported, as displayed in Figure 3a. Spiropyran molecules undergo a ring-opening reaction with a heterolytic C–O bond cleavage, contributing to the formation of zwitterionic merocyanine structures.^46^ To investigate the photoswitchable properties, the time-evolved UV–vis absorption spectra of the spiropyran (SPOH) solutions were measured (Figure 3b,c). Before UV irradiation (365 nm), spiropyran exists in a closed-ring, nonpolar spirocyclic form in its ground state. As shown in Figure 3b, when exposed to UV light over time, the closed spiropyran absorbed photons and underwent a photochemical reaction, leading to the cleavage of the spiro carbon–oxygen bond and the opening of the spiro-ring. As a result, a new absorption peak was observed at a wavelength of ∼425 nm, associated with the π–π* transitions within the conjugated system, indicating the gradual appearance of the open-ring merocyanine-like structures. Upon visible light irradiation, as shown in Figure 3c, the merocyanine-like structures exhibited a continuous decrease in the broad absorption band (∼425 nm) caused by the reversible isomerization of spiropyran.

In previous studies, the photochromic reactions of spiropyran have been mainly investigated in solution systems or bulk states, i.e., the isomerization process of spiropyran was facilitated not in a confined geometry.^53−56^ When the spiropyran polymers were grafted from the AAO membranes, the photochromic properties occurred because the intermolecular packing density was reduced and a sufficient free space was created in the AAO nanochannels, as displayed in Figure 3d,e. Upon UV irradiation, the appearance of the polySP-modified AAO membranes changed to dark purple within seconds, and this change was discernible via UV–vis diffuse reflectance spectra (Figure 3d), which exhibited a characteristic peak at ∼445 nm associated with polyMC-modified AAO membranes. From the UV–vis absorption spectra, the kinetic rate constants of the ring-opening reaction (*k*_p_) for the SPOH solutions and polySP-AAO are estimated to be 0.0488 and 0.0168 s^–1^, respectively, as displayed in Figure S9.

Interestingly, under visible light, the formed polyMC-AAO membranes could be reversibly transformed into polySP-modified AAO membranes with the disappearance of a purple color, as shown in Figure 3e. The reversibility and stability of the photochromic effect of the polySP-modified nanochannels were tested by recording the reflectance at 575 nm after 3 min of UV irradiation and 5 min of exposure to visible light, as illustrated in Figure 3f. The large decrease in reflectivity after the first cycle, observed consistently, indicates the maximum amount of polySP after being stored in darkness. The polySP-modified nanochannels exhibited excellent cycling stability over 20 cycles, attributed to the reversible ring-opening reaction. This stability arose from the separated tethering of the spiropyran polymer onto the nanochannels, a crucial step for stabilizing the spiropyran groups and preventing the aggregation of the merocyanine structures.

**Figure 4:**
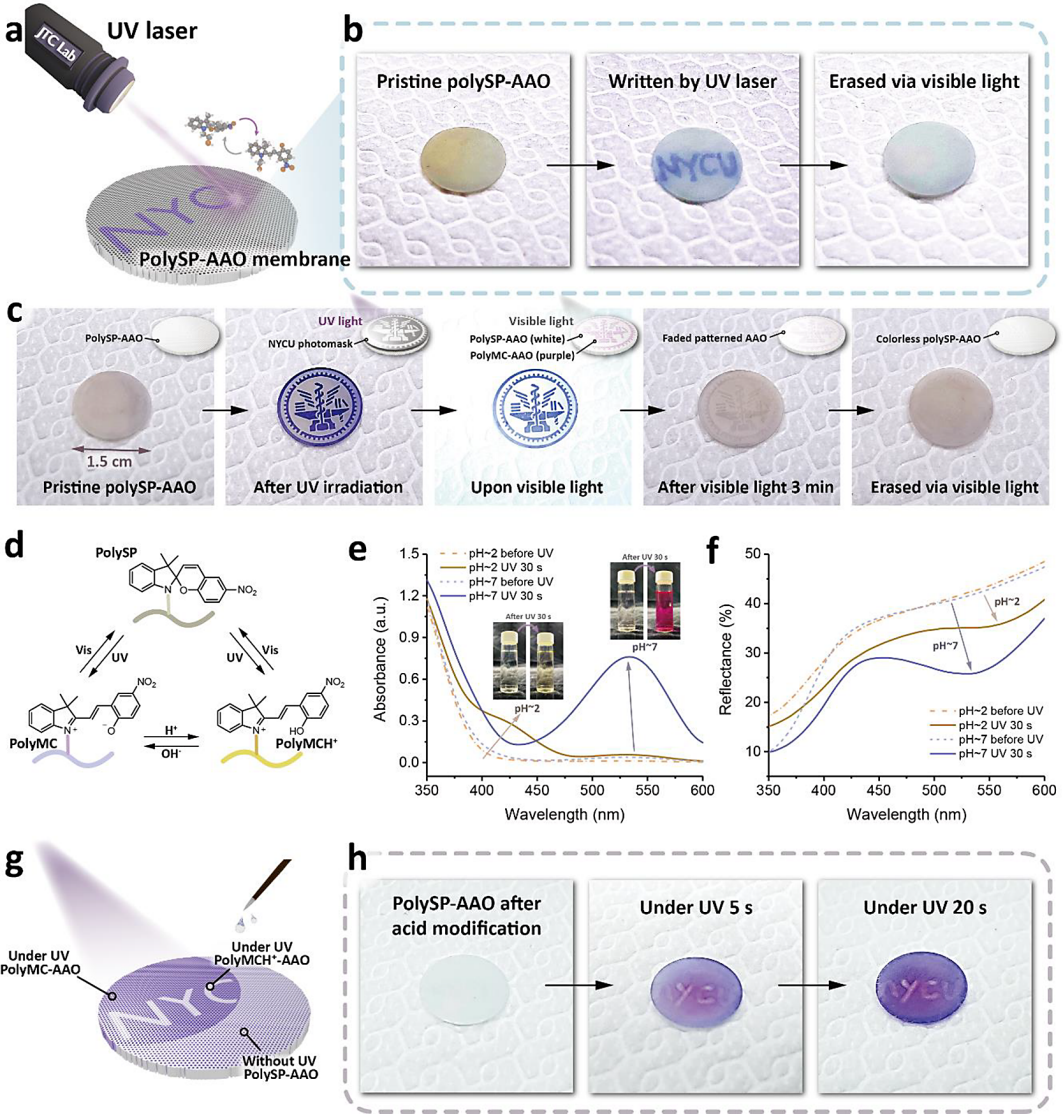
Rewritable and patternable properties of photoswitchable polySP-AAO membranes as anticounterfeiting materials. (a) Schematic illustration of the selective isomerization regions upon short-visible light laser. (b,c) Photographs of the patternable and erasable polySP-grafted AAO (b) written by short-visible light laser and (c) patterned by photomask with NYCU logo. (d) Reversible transformations of SP, MC, and protonated merocyanine (MCH^+^) containing polymers. (e) UV–vis absorption spectra of the SPOH solutions in methanol before and after UV irradiations in different pH values. (f) Reflectance spectra of polySP-modified nanochannels before and after UV irradiations in different pH values. (g) Schematic illustration of the selective acidic treatment upon UV irradiation. Patterns at the regions without UV irradiation are invisible. (h) Photographs of the patternable polySP-grafted AAO for the use as anticounterfeiting materials.

For the phototactic response of green algae, the sensitivity of their eyespots plays a crucial role in tuning their moving direction. In the same vein, by exposure of the polySP-AAO membranes to a short-visible light laser (405 nm, 0.5 mm light spot), selective ring-opening isomerization could be achieved, as displayed in Figure 4a. Figure 4b presents the photographs of patternable and erasable polySP-grafted AAO. Before shining with a short-visible light laser, the polySP-AAO membranes were pale yellow, indicating the spiropyran structures in the polymers. Upon short-visible light laser, only tiny regions were induced to undergo ring-opening isomerization, which was written on a much smaller scale according to the light spot of the short-visible light laser. After visible light irradiation, the patterns on the polySP-AAO membranes were erased by reversible isomerization, resulting in both invisible patterns and colorless surfaces. The reversible photochromic effect on the selective regions, which was more stable by polymer grafting modifications, demonstrated patternable membranes with precise light control. By applying a photomask to the polySP-AAO during UV irradiation, the NYCU logo pattern became clearly visible on a relatively small scale (∼1.5 cm) and could be erased by shining with visible light, as displayed in Figure 4c.

In addition to the photocontrollable properties of SP moieties, selective chromism can also be investigated due to configuration transformation in acidic/basic conditions, as discussed in previous works.^46,52^Figure 4d presents the interconversion of SP, MC, and protonated merocyanine (MCH^+^) containing polymers. Under acidic conditions, the MC isomer could be converted to a light yellow MCH^+^ form, as displayed in Figure 4e. Taking the different configurations of SP under acidic conditions, we further investigated the UV–vis diffuse reflectance of polySP-AAO, as shown in Figure 4f. The polySP-AAO membranes with acidic treatment presented a color change from white to light yellow (polyMCH^+^-AAO) upon UV irradiation, whereas the polyMC-AAO membrane transformed to light purple.

The possibility of loading information in polySP-AAO and unlocking the data by UV light was explored through selective acidic treatments. To address this aspect, the surfaces of polySP-AAO membranes were patterned by 0.1 M HCl_(aq)_ on selective regions, which was invisible without shining UV light, as shown in Figure 4g. We hypothesized that the hidden patterns could appear under UV light based on different configurations of polyMC and polyMCH^+^. Figure 4h presents photographs of the patternable polySP-grafted AAO membranes. Initially, the membranes stayed white color. Upon UV irradiation for a few seconds, the words “NYCU” regions remained white color because of the relative acidic condition, inducing the presence of the polyMCH^+^, whereas the other regions still presented a color change to purple (polyMC). The controllable and reversible photochromic effect in both light and pH stimuli, more stable with polymer grafting modifications, demonstrated potential applications in information encryption and anticounterfeiting.

**Figure 5:**
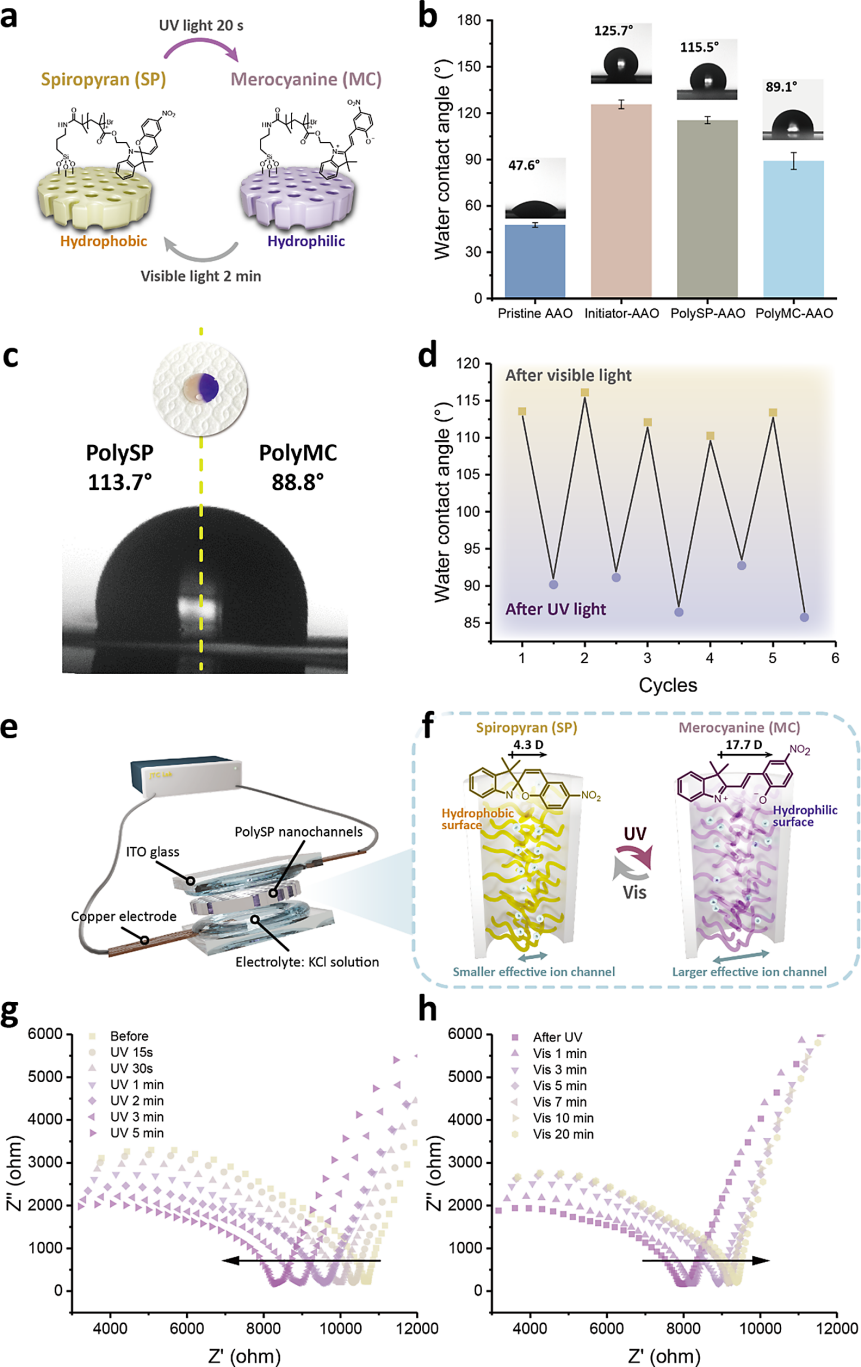
Photoswitching of the wettabilities in photoswitchable ion nanochannels. (a) Schematic illustration of the reversible wettabilities by polySP-modified nanochannels. (b) Plots and images of water contact angles of the pristine AAO, initiator-immobilized AAO, polySP-grafted AAO, and polyMC-grafted AAO. (c) Asymmetric water contact angle of a sample that is shone with UV light on half of the region. (d) Plots of water contact angles upon UV and visible light irradiations in different cycles. (e–h) Photoswitching of the electrochemical properties in the polySP photoswitchable ion nanochannels via controllable wettabilities. (e) Illustration of the electrode configuration for the electrochemical measurements. (f) Working mechanism and internal structure of the polySP-AAO. (g,h) Electrochemical impedance spectra of the polySP-AAO: (g) upon UV and (h) visible light irradiations.

To our knowledge, surface properties (i.e., surface charge, wettability, and roughness) play crucial roles in influencing ion transport in nanochannels.^57,58^ As a result, to further understand the photoswitchable wettability changing the behavior of the polySP-AAO, time-dependent water contact angle (WCA) measurements were recorded. As shown in Figure 5a–d, each static contact angle measurement was measured by dropping a water droplet (4 μL) onto the surfaces of the AAO membranes. Figure 5a illustrates the wettability change during the ring-opening reactions of the polySP-grafted AAO membranes. In the initial state, the spiropyran polymer was typically hydrophobic because of the nonpolar closed-ring structure and the hydrocarbon structure of the polymer. When exposed to UV light, spiropyran underwent an isomerization to form an open-ring merocyanine structure. This process involved breaking of the spiro carbon–oxygen bond, resulting in a more polar and hydrophilic structure.

Figure 5b presents the water contact angles of the modified AAO membranes in every step. Compared with the pristine AAO membranes, the WCA of the initiator-AAO increased from 47.6 to 125.7°, contributing to the nonpolar nature of the alkyl chains and the limited interaction of the bromine end. After the polySP grafting process, the WCA was recorded as 115.5°, which was related to the nonpolar close-ring structure, polymer chains, and surface roughness, suggesting the Cassie–Baxter state of the water droplet.^59^ While shining with UV light for 20 s, the spiropyran polymers in the polySP-AAO transformed to a purple-colored merocyanine form with a WCA at 89.1°, which revealed the presence of polar functional groups and charge distribution in the ring-opening structure, indicating the Wenzel state of the water droplet.^59^ Compared with previous works on the wettability of SP polymers,^60,61^ the higher hydrophobic surfaces observed in our study were due not only to the SP polymer with longer alkyl chains but also to the nanoporous structures of the AAO membranes. When placing the polySP-AAO membranes in MeOH, which is a good solvent for SPMA structures (also for PMMA), the polymer can be swollen but not dissolved, because of the grafting and stabilizing on the nanochannels. When placing the polySP-AAO membranes in water or electrolyte aqueous solution, the swelling degrees of the SP polymers could be less than those in MeOH.

To further demonstrate the selective photoinduced wettability of the polySP-AAO membranes, UV light was irradiated on a specific region to create an AAO membrane with half polySP grafting and half polyMC grafting, as shown in Figure 5c. A water droplet was placed at the boundary, and the WCA was observed at 113.7 and 88.8° at the same location, confirming the wettability of the polySP and polyMC structures. To examine the reversibility of the polySP-AAO membranes, the photoinduced WCA experiments were repeated 5 times without interruption, as presented in Figure 5d. The cyclic results indicated that the polySP-AAO membranes possessed not only fast response (within seconds) but also good repeatability, which is comparable to other reported fast-response ion channels.^62,63^ Compared with the previous study,^44^ the water flux of our system was assumed to be affected because of the ring-opening reaction and the exhibition of surface charges of the MC polymer; however, the water flux could not be obvious because of the lower WCA difference.

The precise photoinduced wettability of the polySP-grafted AAO membranes provides an exciting opportunity for tuning the ion conductivity via reversible ring-opening isomerization. As reported in previous works,^64,65^ the wettability change of spiropyran-based materials could be enhanced by adding ions, indicating that the control of ion conductivity with wettability change can be further improved by adding a low-concentration electrolyte solution. Figure 5e illustrates the setup of the electrochemical measurements. By application of a voltage at 1 V, the impedance change was recorded upon light stimulation. A polySP-grafted AAO membrane was sandwiched between two ITO glasses and two Teflon spacers, which were used as containers of electrolytes (0.3 mM KCl solution at pH = 7). The ITO glasses were connected with copper electrodes, and the working area was 0.92 cm^2^. Figure 5f illustrates the working mechanism of the photoswitchable ion nanochannels in polySP-grafted AAO membranes. While polySP-grafted AAO membrane absorbed the UV light, the heterolytic C–O bonds in the spiropyran polymers were cleaved, forming the zwitterionic merocyanine structures. The hydrophilic surfaces of the highly polar merocyanine structures encouraged the formation of a stable hydration layer around the ions, enhancing ion transportation between different sides of the nanochannels.^20,66^ After the UV light was turned off, the merocyanine structures gradually converted back to spiropyran by ring-closing isomerization, providing a water-exclusion environment that can hinder ion mobility.

To examine the photoinduced ion gating behaviors of the polySP-grafted AAO membranes, electrochemical impedance spectroscopy (EIS) analysis was conducted, as presented in Figure 5g,h. A polySP-grafted AAO membrane with pore sizes of ∼100 nm was measured over time upon UV irradiation and visible light at room temperature. During UV irradiation, the overall impedance magnitudes (*Z*) gradually decreased, while relatively higher impedance magnitudes were observed when irradiated with visible light.

**Figure 6:**
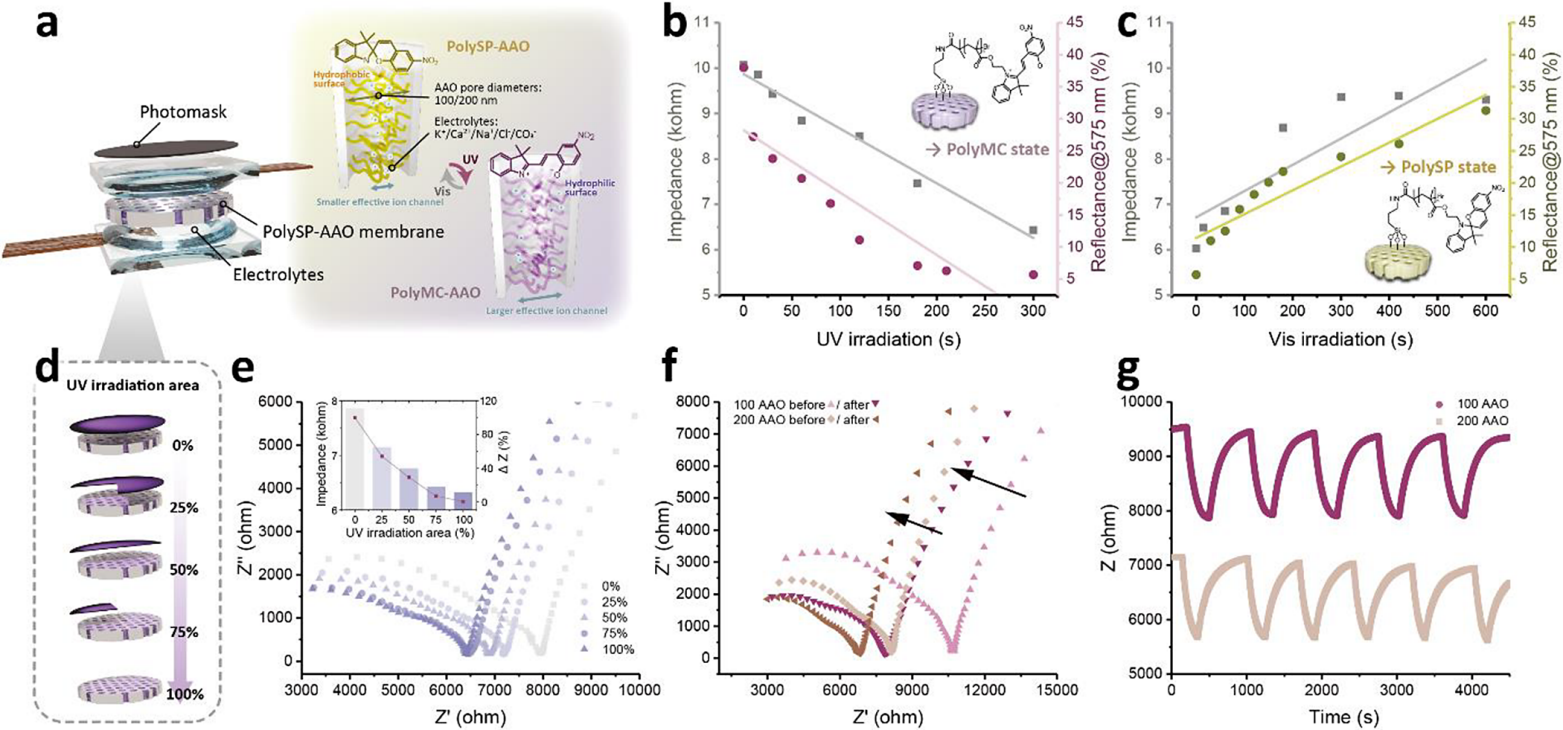
Photoswitching of the electrochemical properties in the polySP-AAO using different parameters. (a) Illustration of the electrode configuration, parameters, and mechanism. (b,c) Summarized plots of the impedance and reflectance changes: (b) under UV and (c) visible light irradiations for different times. (d,e) Controllable impedances with different UV irradiation areas: (d) graphical illustration of the polySP-AAO under different photomasks and (e) electrochemical impedance spectra and summarized plot of the polySP-AAO with different UV coverages. (f,g) Electrochemical impedance spectra of the polySP-AAO with different pore sizes for (f) single cycle and (g) different cycles.

Several experimental parameters of polySP-AAO were also discussed, such as photomasks, AAO pore diameters, and electrolytes, as shown in Figure 6a. By fitting the Nyquist plots, the impedances and the detected reflectances of the polySP-grafted AAO membranes were calculated and are summarized in Figure 6b,c. Intriguingly, the impedance evolution and isomerization kinetics of the polySP/MC-grafted AAO membranes demonstrated a comparable evolution, indicating their high correlation.

To deepen the understanding of the characteristics of polySP in the nanochannels, different irradiation areas upon UV treatment were applied, causing photoisomerization to varying degrees and leading to controllable impedance in the polySP-AAO nanochannels. Photomasks with different sizes (Figure 6d) were placed on the electrode sample to generate varying UV light coverages. Under UV light, the impedances gradually decreased with an increased UV light coverage, as shown in Figure 6e. Different pore sizes of AAO membranes were tested, as displayed in Figure 6f. It should be noted that the AAO membrane did not contribute to the ion conductivity, which means that the modified nanochannels were the only conducting regions in the samples. Owing to the higher volume of the ion transport pathway, the polySP-AAO nanochannels with pore sizes of 200 nm revealed lower impedance based on their higher porosity at ∼44.7%, in contrast to the lower porosity at ∼36.7% for 100 nm AAO nanochannels. Furthermore, compared with KCl electrolytes, the impedances were relatively lower in the nanochannels filled with Na_2_CO_3_ and CaCl_2_, caused by different amounts and ion transport of the ions, as presented in Figure S10 and Table S2, respectively.

In previous studies, the photofatigue effects observed in spiropyran-based functional materials often stem from the aggregation of individual merocyanine isomers, which have constrained the practical use in real-world applications.^67,68^ To examine the stability of the polySP photoswitchable ion nanochannels, cyclic measurements of polySP-AAO nanochannels with different pore sizes were performed uninterruptedly, as shown in Figure 6g. Similar to the results tracked by UV–vis reflectance, there was no noticeable decrease in the photocontrolled impedance after repeating for several cycles. The calculation method and relative ionic conductivity cyclic data were also shown in Note S1 and Figure S11, respectively. The phenomenon was ascribed to the fact that polymer structure not only provided a high density of the photochromic spiropyran groups but also prevented the aggregation of the merocyanine groups even in nanoscale confined environments, which was a noteworthy result and possible solution to the photofatigue effect of spiropyran-based functional materials.

## Conclusions

In this study, we demonstrated the design and fabrication of photoswitchable ion nanochannels, inspired by natural ChRs, by using polySP-grafted AAO membranes. The integration of SP molecules into these nanochannels, achieved through SI-ATRP, enabled the creation of a responsive system capable of modulating its ionic conductivity and hydrophilicity in response to light stimuli. The chemical composition and functionality of these channels were further substantiated by XPS, SEM, and TGA analyses. The effective mimicry of ChR ion channels was demonstrated by the reversible ring-opening isomerization of spiropyran groups under UV irradiation. This transformation, observable both macroscopically and at the molecular level, endowed the nanochannels with the ability to switch between hydrophobic and hydrophilic states, thereby modulating the ion transport efficiency. The patternable and erasable polySP-grafted AAO membranes based on the controllable and reversible photochromic effect demonstrated potential applications in anticounterfeiting. The polySP-modified photoswitchable ion nanochannels present an efficient approach to controlling ionic transport and offer a promising platform for future developments in sensing, controlled ion transport, and optical data storage.

## Methods

### Synthesis of PolySP-Grafted AAO Nanochannels

Before SI-ATRP, pristine AAO membranes were modified with an H_2_O_2_ solution, followed by immobilization of aminopropyl-triethoxylsilane (APTES) and 2-bromobutyryl bromide as ATRP-initiators. The polySP-grafted AAO nanochannels were obtained by SI-ATRP using monomer SPMA (60 mg), CuBr (2.9 mg), and an ATRP initiator-grafted AAO membrane in a N_2_ atmosphere for 1 h. After the SI-ATRP, the polySP-grafted AAO membranes were washed with ethanol several times and dried under vacuum before further applications. The detailed synthetic process is presented in Supporting Information.

### Photochromic Experiments and Hydrophilicity Tests of the PolySP-Grafted AAO Nanochannels

A spiropyran-containing polymer (polySP) grafted AAO membrane was first irradiated with a UV light source (365–375 nm, 300–700 mW) for 20 s, forming the merocyanine-containing polymer (polyMC) grafted AAO membrane. To change the merocyanine state back to the spiropyran state, the polyMC-grafted AAO membrane was placed under visible light for 2 min. The solvent used for UV–vis measurements was methanol at an SP concentration of 1 mg/5 mL. For the photochromic experiment, the samples were illuminated by a three-wavelength light source containing UV and vis light (Flaming Fire XP-G; visible light: 350 lm; UV light: 365–375 nm, 300–700 mW). For the hydrophilicity tests, 4 μL of water droplets were dropped on the surfaces of the samples. Asymmetric water contact angles were tested by shining half of the region of the sample with UV irradiation.

### Electrochemical Impedance Test of the PolySP-Grafted AAO Nanochannels

The EIS testing was demonstrated by placing a polySP-grafted AAO membrane between two torus-shaped Teflon spacers and two ITO glasses. By filling the KCl solution (0.02 mg/mL) into the middle hole of the spacers on both sides, the nanopores were infiltrated with electrolytes. For the EIS testing, the amplitude of the applied voltage was 0.5 V, and the frequency range was set at 1 Hz to 1 MHz. The fitting analyses were performed using electrochemical fitting software (ZView2).

## Supporting Information Available

Synthetic scheme, ^1^H NMR, and FT-IR spectra of spiropyran derivatives; materials, methods, and experimental details; side-view SEM images of the pristine and polySP-AAO membranes; EDS mapping, atomic ratios, and line scan data of a polySP-AAO membrane; plots of data from the UV absorption and reflection for the kinetic rate constants; and EIS data of the polySP-AAO upon UV irradiation with different electrolytes and pore diameters (PDF)
