# Supplementary material for: Illuminating Biomimetic Nanochannels: Unveiling Macroscopic Anticounterfeiting and Photoswitchable Ion Conductivity via Polymer Tailoring
Source: ACS Nano. 2024 Sep 20;18(39):26948–60. doi: 10.1021/acsnano.4c08801 (PMC11447919; doi:10.1021/acsnano.4c08801)
Supplement: Supplementary file 1 — nn4c08801_si_001.pdf [file nn4c08801_si_001.pdf]

Supporting Information

Illuminating Biomimetic Nanochannels: Unveiling Macroscopic Anticounterfeiting and Photoswitchable Ion Conductivity via Polymer Tailoring

Yi-Fan Chen,[1] Vaishali Pruthi,[2] Lin-Ruei Lee,[1] Yu-Chun Liu,[1] Ming-Hsuan Chang,[1] Patrick Théato,[2,3]* and Jiun-Tai Chen[1,4]*

[1]Department of Applied Chemistry, National Yang Ming Chiao Tung University, 300093 Hsinchu, Taiwan

[2]Institute for Chemical Technology and Polymer Chemistry (ITCP), Karlsruhe Institute of Technology (KIT), Kaiserstraße 12, D-76131 Karlsruhe, Germany

[3]Soft Matter Synthesis Laboratory Institute for Biological Interfaces III, Karlsruhe Institute of Technology (KIT), Hermann-von-Helmholtz-Platz 1, D-76344 Eggenstein-Leopoldshafen, Germany

[4]Center for Emergent Functional Matter Science, National Yang Ming Chiao Tung University, 300093 Hsinchu, Taiwan

Experimental Section

Materials

2,3,3-Trimethyl-3H-indole, 2-hydroxy-5-nitrobenzaldehyde, and methacryloyl chloride were purchased from Sigma-Aldrich and used as received. 2-Bromoethanol was obtained from Tokyo Chemical Industry. (3-Aminopropyl)triethoxysilane (98%) was obtained from Alfa Aesar. Initiator 2-bromoisobutyryl bromide ($C_4H_6Br_2O$, 98%) and copper (I) bromide (CuBr, 98%) were purchased from Sigma-Aldrich. *N, N, N', N'', N''*-pentamethyldiethylenetriamine (PMDETA, 98%) was bought from Nova Materials. Hydrogen peroxide (35 wt %) was obtained from Honeywell. Potassium chloride (KCl, 99%) was bought from Union Chemical. *n*-Hexane (99.5%) and dry tetrahydrofuran were purchased from Fisher Scientific. Acetone (99.5%) was obtained from Echo. All the chemicals were used without further purification. AAO membranes (diameter~13 mm, thickness~60 μm) were purchased from Whatman with different nanopore sizes. Microscope glass slides were obtained from Dogger Scientific (DGS).

Synthesis of Photoresponsive Spiropyran Molecules and Monomers

The spiropyran derivative (2-(3',3'-dimethyl-6-nitro-3'H-spiro[chromene-2,2'-indol]−1'-yl)-ethanol, SPOH) was synthesized from 2,3,3-trimethyl-3H-indole, based on our previous study with modifications,[1] as presented in Figures S1-S2. For the polymerization inside the nanochannels, polymerizable methacrylate groups were attached on SPOH, as shown in Figures S3-S4. Fourier-transform infrared (FTIR) results of SPOH and SPMA were presented in Figure S5.

Synthesis of Spiropyran Derivative (SPOH)

SPOH was prepared by following previous studies with some modifications. The synthetic scheme of spiropyran derivative (SPOH) is shown in Figure S1. First, 2,3,3-trimethyl-3H-indole (2.6 g, 16 mmol)

and 2-bromoethanol (2.46 g, 20 mmol) were mixed in 20 mL of acetonitrile. The solution was refluxed for 24 h at 85 °C under N_2. After cooling down to room temperature, the solvent was removed by rotary evaporation. The residue was suspended in 30 mL of hexane, followed by sonication and filtration. Then, the obtained intermediate, bromide salt, was crystallized by chloroform.

The obtained bromide salt (2.93 g, 10 mmol) was dissolved in 50 mL of 0.3 M $KOH_{(aq)}$. After stirring at room temperature for 15 min, the solution was extracted with diethyl ether. The organic phase was concentrated, and an oxazole derivative was obtained.

The oxazole derivative (9,9,9a-trimethyl-2,3,9,9a-tetrahydro-oxazolo [3,2-a]indole, 1.09 g, 5 mmol) and 2-hydroxy-5-nitrobenzaldehyde (1.25 g, 7.5 mmol) were added in 10 mL of ethanol. The solution was refluxed for 24 h at 65 °C under N_2, followed by cooling to room temperature. The dark purple solid, SPOH, was obtained after the solution was distilled by rotary evaporation.

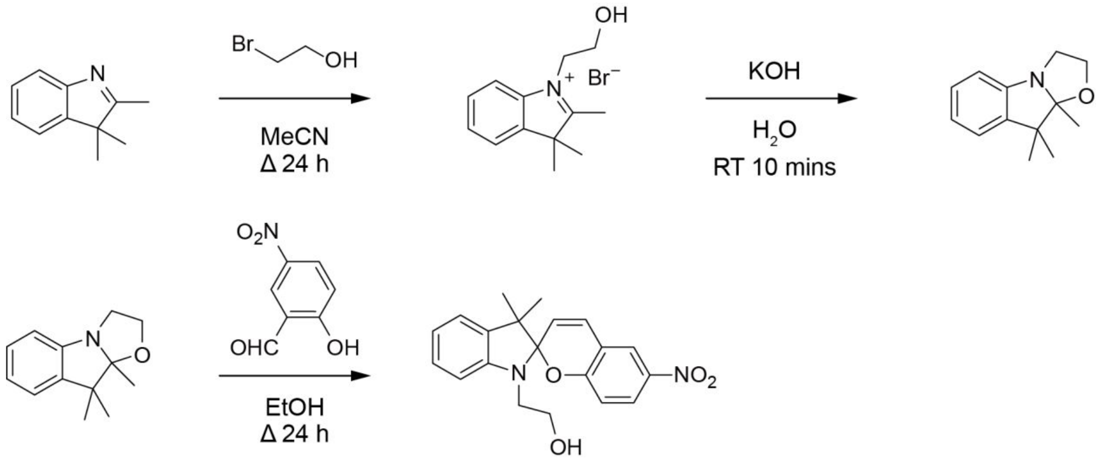

Figure S1. Synthetic scheme of the spiropyran derivative (SPOH).

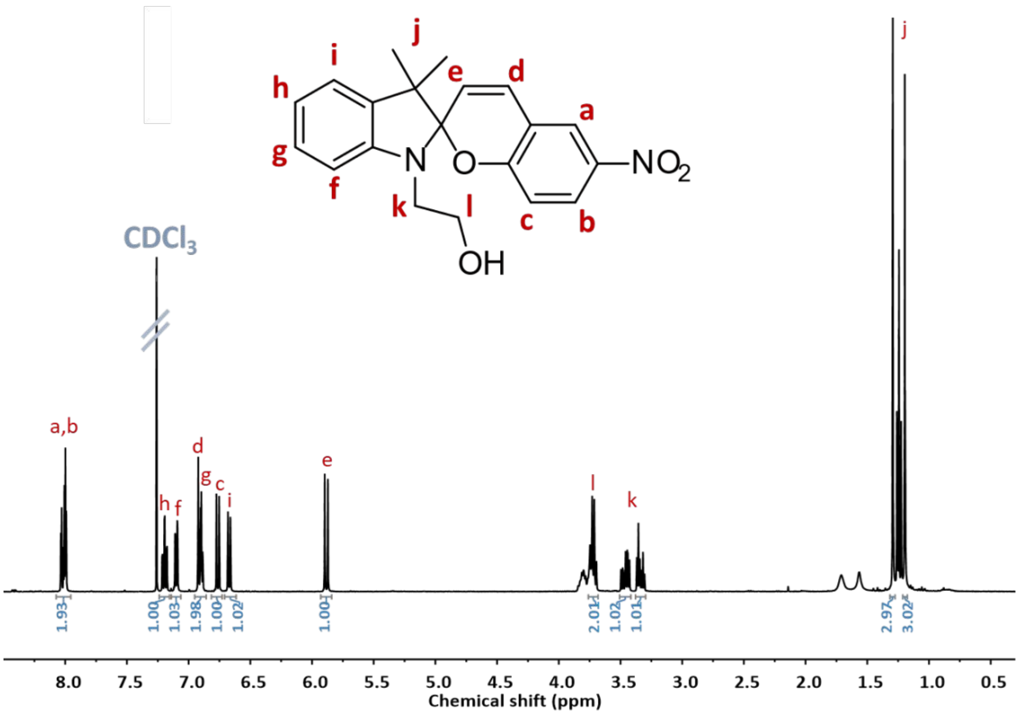

Figure S2. ^{1}H NMR spectrum (400 MHz, $CDCl_3$, 298 K) of the 2-(3',3'-dimethyl-6-nitro-3'H-spiro[chromene-2,2'-indol]-1'-yl)-ethanol (SPOH).

Synthesis of Spiropyran Methacrylate (SPMA)

SPOH (1.05 g, 3 mmol), methacryloyl chloride (0.784 g , 7.5 mmol), Et_3N (0.758 g, 7.5 mmol), and CH_2Cl_2 (40 mL) were loaded into a round-bottom flask at 0 °C. The reaction was stirred for 10 h, followed by filtration and extraction ($NaHCO_3$:water = 1:1). The organic layer was dried by $MgSO_4$ and then concentrated under reduced pressure to get the crude compound. The obtained crude (purple solid) was purified using column chromatography by eluting from ethyl acetate/hexane = 1:1. After crystallization in 95% ethanol, pure SPMA was obtained.

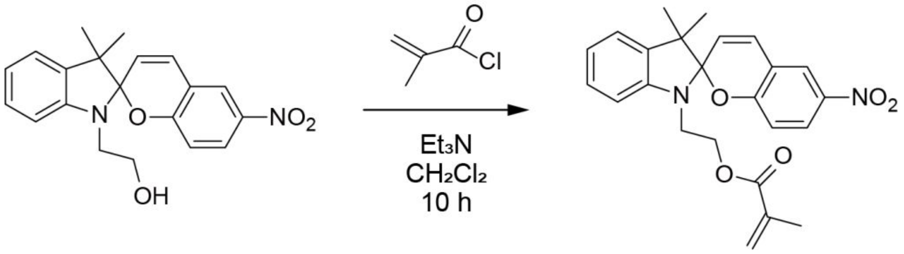

Figure S3. Synthetic scheme of the spiropyran methacrylate (SPMA).

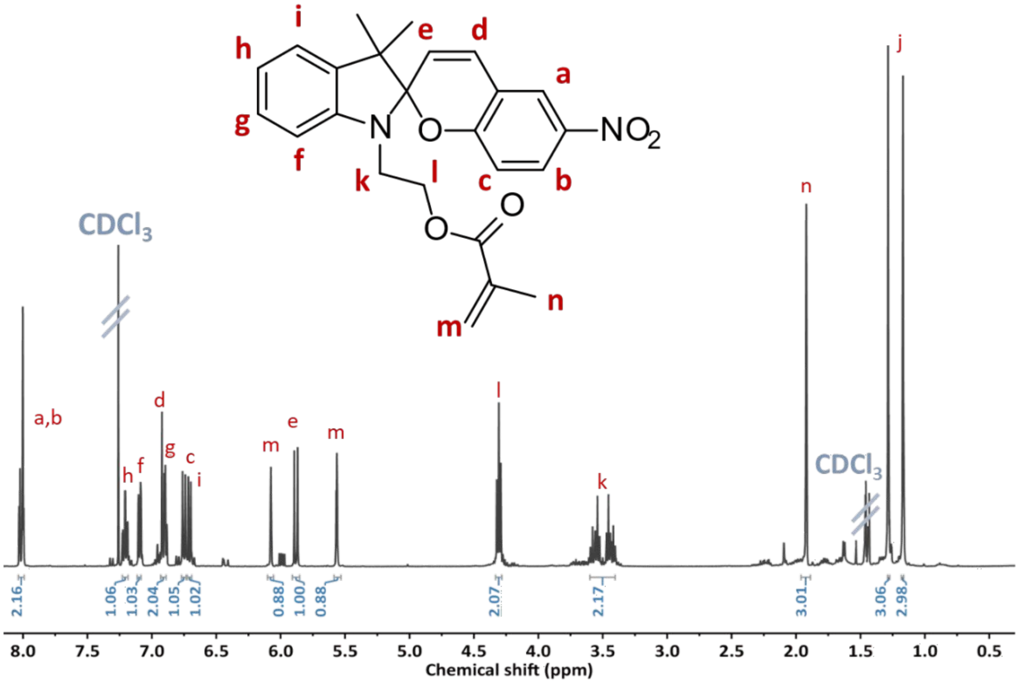

Figure S4. ^{1}H NMR spectrum (400 MHz, $CDCl_3$, 298 K) of the spiropyran methacrylate (SPMA).

Synthesis of ATRP Initiator-Grafted AAO Nanochannels

To enhance the density of the OH group on the surface of AAO membranes, pristine AAO membranes were immersed in 34 wt % H_2O_2 solution for 30 min and then cleaned with deionized water and ethanol several times. After the H_2O_2 pretreatment, the AAO membranes were placed in a solution of 5% aminopropyl-triethoxylsilane (APTES), to enable silanization of the interior surface of the AAO membranes resulting in APTES-grafted AAO membranes expressing of NH_2-groups at the surface. After immersion for 30 min, the AAO membranes were rinsed with ethanol several times and dried under reduced pressure. The APTES-grafted AAO membranes were placed in a 25 mL round bottle containing 4 mL triethylamine and 10 mL dichloromethane. After N_2 purging for 15 min in an ice water bath, the surface was reacted with 2-bromobutyryl bromide yielding an ATRP surface initiator. The ATRP initiator-grafted reaction was placed in the ice bath for 1 h and reacted at room temperature for 12 h. The sample was then washed by dichloromethane and dried in a vacuum.

Synthesis of PolySP-Grafted AAO Nanochannels

The polySP-grafted AAO nanochannels were obtained by SI-ATRP. For this, the monomer SPMA (60 mg), CuBr (2.9 mg), and ATRP initiator-grafted AAO membranes were placed in a 25 mL round bottle. Then, 6 mL of dry THF and 1.5 μL of EBiB were injected into the bottle. After being kept in an N_2 atmosphere for 1 h, the catalyst PMDETA (12.8 μL) was injected quickly. Subsequently, the reaction was kept in an oil bath at 60 °C for 24 h. After the SI-ATRP, the polySP-grafted AAO membranes were washed with ethanol several times and dried in a vacuum.

Structure Characterizations and Analyses

To investigate the surface morphologies of the samples, a scanning electron microscope (SEM, JEOL JSM-7401F) was used at an acceleration voltage (5 kV). The polymer films and AAO membranes were coated with platinum (4 nm) before performing the SEM measurements. To confirm the chemical structures, Fourier-transform infrared (FTIR) analyses were examined using an IR spectrometer (PerkinElmer Spectrum One). To record the photoinduced ring-opening reaction of spiropyran molecules, time-evolved UV-vis absorption spectra were conducted by a Hitachi U4100 ultraviolet-visible (UV-vis) spectrometer. To conduct thermal stability analyses, a TGA 55 (TA Instruments) was applied at a temperature range of 100−650 °C (heating rate: 10 °C/min) under N_2 atmosphere. To examine the hydrophobicity, water contact angles (WCA) were measured by a goniometer (FTA 125, First Ten Ångstroms) under ambient conditions with dropping water droplets (4 μL) on the surfaces of the samples. Both WCA measurements in single and cycle tests were obtained at static state, in which samples were dried in a vacuum oven in between. To measure the electrochemical properties, electrochemical impedance spectroscopy (EIS) measurements were collected by CHI6000 (CHI instruments). By sandwiching the polySP-grafted AAO membrane between two spacers and ITO glasses, the impedances of samples were measured over a frequency range of 1 Hz to 1 MHz under UV and visible light irradiations. For the short-visible light laser, an excitation light source (Laserland, 405 nm) was applied for the patterning applications (output powers: 50 mW, laser beam diameter: ~0.5 mm, and laser power density: 78 mW/cm^2).

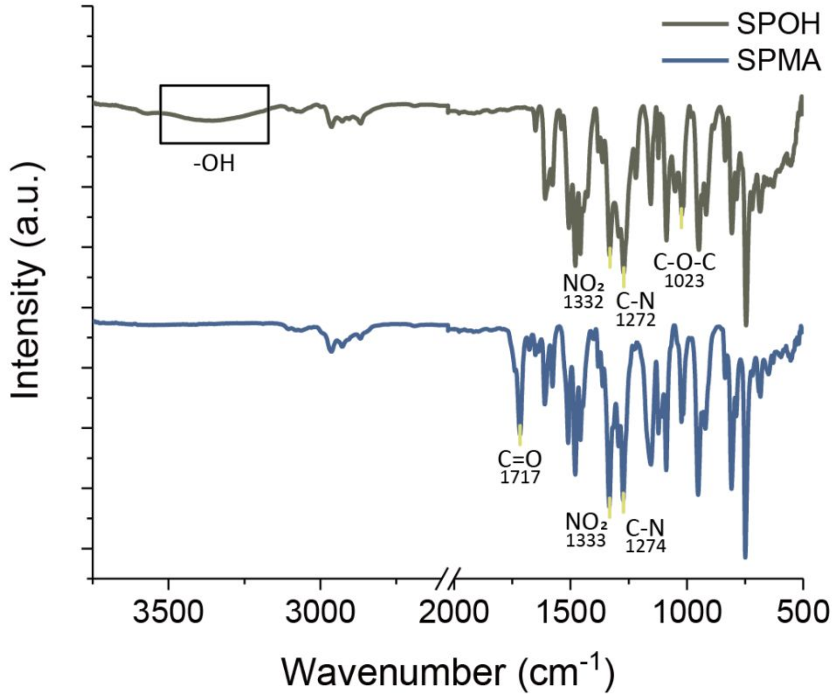

Figure S5. FTIR data of SPOH and SPMA.

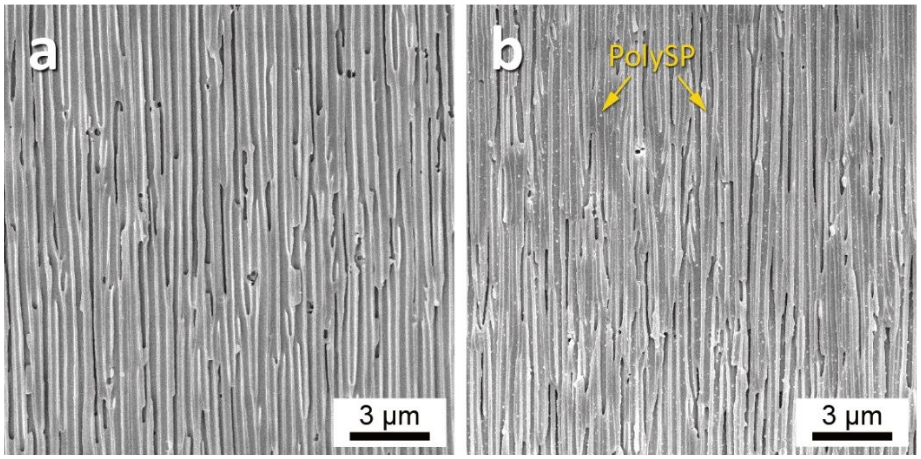

Figure S6. Side-view SEM images of (a) the pristine and (b) polySP-modified AAO membranes.

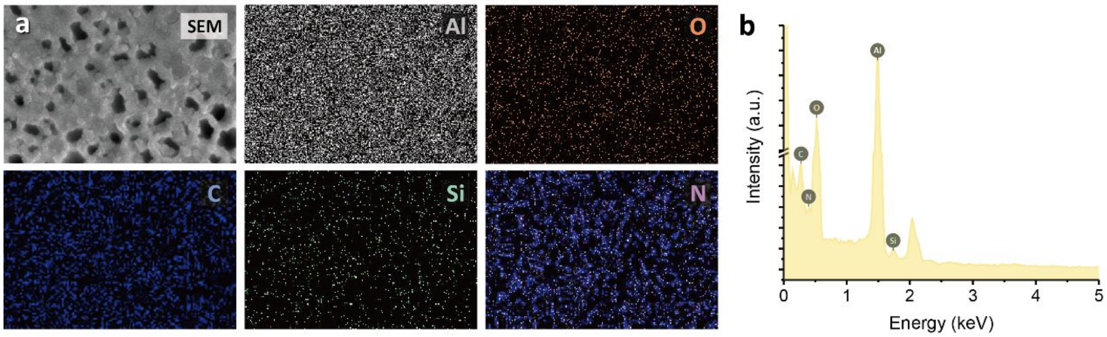

Figure S7. EDS mapping of a polySP-AAO membrane. (a) Top-view SEM image and mapping data of Al, O, C, Si, and N. (b) EDS data of the polySP-AAO membrane.

Table S1. Atomic ratios of the polySP-AAO membranes

PolySP-AAO membranes	Atomic %
**C**	18.68
**N**	7.22
**O**	60.23
**Al**	12.81
**Si**	0.25

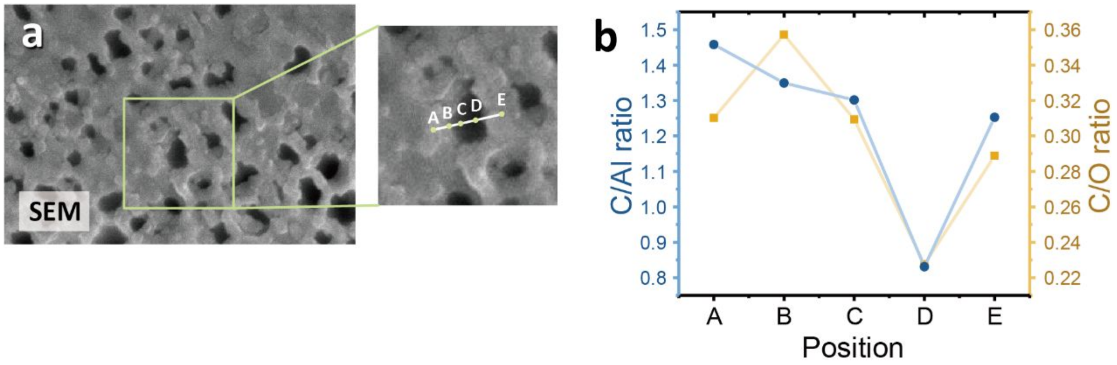

Figure S8. EDS line scan data of a polySP-AAO membrane. (a) Top-view SEM image and line scan data (A-E). (b) EDS C/Al and C/O ratios of the polySP-AAO membrane.

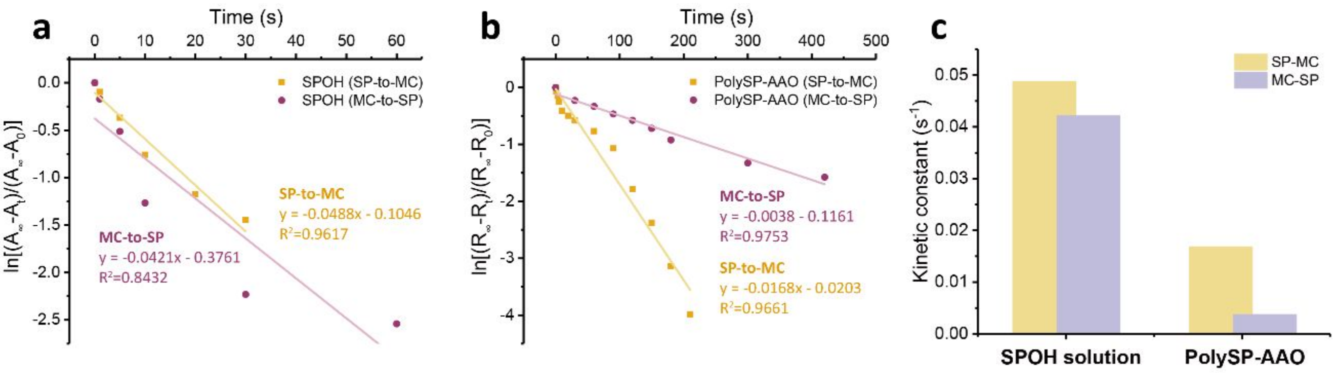

Figure S9. Plots of data from the UV absorption and reflection to obtain the kinetic rate constants: (a) SPOH solution and (b) polySP-AAO membrane. (c) Comparison and summary of the kinetic rate constants.

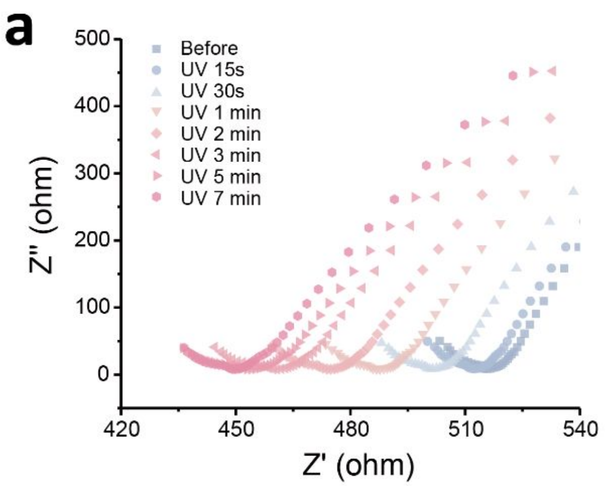

Figure S10. Electrochemical impedance spectra of the polySP-AAO upon UV irradiation: (a) Na_2CO_3 and (b) $CaCl_2$.

Table S2. Summarized table of the impedance, ionic conductivity, and percentage of reduced impedance of the polySP-AAO nanochannels with different electrolytes

**Electrolytes**	**Impedance (nm)**		**Conductivity (S/cm)**		**$\Delta Z/Z_{max}$ (%)**
**Under UV**	Before	After	Before	After	-
**KCl**	8.14E+03	6.78E+03	2.5E-04	3.0E-04	16.71
**Na_2CO_3**	5.18E+02	4.50E+01	3.9E-03	4.5E-03	13.12
**$CaCl_2$**	4.07E+02	3.41E+02	5.0E-03	6.0E-03	16.22

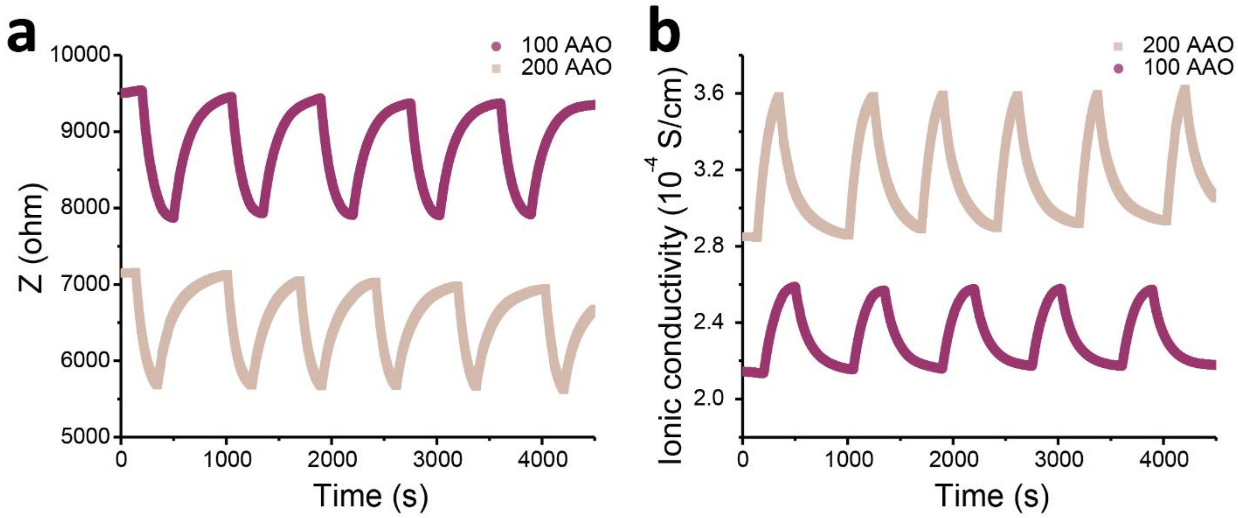

Figure S11. (a) Electrochemical impedance and (b) ionic conductivity cyclic data of the polySP-AAO with different pore diameters of 100 and 200 nm AAO.

Note S1. The calculation of ionic conductivity

The ionic conductivity of the polySP-AAO nanochannels is calculated by the following equation:

$$\sigma = \frac{l}{R_b \times A}$$

where σ is the ionic conductivity (S/cm), l is the thickness of the sample (cm), R_b is the bulk resistance obtained from Nyquist plots, A is the contact area of the sample.
